# Alaska: Its Neglected Past; Its Brilliant Future

**Published:** 1898-09

**Authors:** 


### BOOK NOTICES.

ALASKA, ITS NEGLECTED PAST; ITS BRILLIANT FUTURE. By Bushrod Washington James, M. D. Philadelphia. The Sunshine Publishing Company. 1897.

This interesting book has been before the public for six months. It is the work of our well known professional brother, Dr Bushrod W. James, author of the poem on Alaska's legends, entitled "Alaskana," written after the style of Longfellow's poem, "Hiawatha." This poem was noticed in THE HOMŒOPATHIC PHYSICIAN for January, 1893, page 60. The work now under notice is intended to supply the demand for definite information concerning Alaska, now that it has become so attractive to the whole civilized world by reason of the gold fields. The author expects it to be used as a companion by those who are going to those tempting fields of untold wealth. It contains some good charts of the region and many good photographic plates of the land and water scenery of that desolate country.

The author discourages the tendency of the mining population to cross the boundary of the United States territory and enter the country of the British flag when they might be quite as successful by remaining within the borders of the land that claims the Stars and Stripes. The author exclaims: "Why will American citizens risk their lives and their all in prospecting the Klondyke and other streams on British territory, when those waters are really only branches of the grand trunk that belongs within entirely undisputed United States property? Like children trampling beauteous blossoms under foot while reaching for others beyond, so are the miners of the United States when they clamber over the mountains and row through the waters of their own land to reach that of another nation, when if the country through which they travel were searched and prospected as eagerly as they intend to investigate the Klondyke region they will surely find sufficient riches to pay them for stopping under the flag whose protection is theirs by right, and no international entanglements or suits for mining claims would be likely to ensue."

After dwelling upon the "needs of Alaska" from a legislative point of view, the author tells you how to reach Alaska.

It appears that the gold was actually discovered by the Indians, who found the metal in the sands of the creek near Auk glacier, about October, 1880.

The author then gives the narrative of his own trip through this wonderful country; his narrative being fully illustrated, as before stated, with photographic plates.

In the course of his travels the author visits some gold mines. He says: "Think of a gold-bearing quartz vein four hundred feet wide, the Bear's Nest vein one hundred feet wider; or one six hundred feet wide, as the Lorena mine ledge on Admiralty Island! There is a feeling akin to the pride of proprietorship in the hearts of all true-born Americans when we are told that there is sufficient gold in sight to pay the price of the Territory two or three times over."

Descriptions of the scenery of the glaciers, of the birth of an iceberg, and poetical reflections upon the grandeur and the beauty of the prospect are indulged. Then comes a description of Sitka, and then an intensely interesting description of the Fur Seal Islands, with a condensed statement of how the fur is prepared. Descriptions of the Aleutian Islands follow, always in a poetic vein.

Our Alaskan interests are treated and a plea is entered for an alliance between the United States, Russia, Japan, and China for the protection of the seal fisheries and other interests of that region. Then comes a demand for the settlement of the boundary question between the property of the United States and that of Great Britain. The alliance of the United States with Russia, China, and Japan to protect the seal fisheries is again urged, and then there follows an analysis of the seal catch.

The crime of pelagic sealing is exposed in all its horrors, and the iniquity of persisting in it and refusing to accept any modification of this method of sealing or of considering its barbarity is warmly dwelt upon, and the people of the United States appealed to for measures that will effectually stop it. The author enters into a discussion upon international law as affecting the Bering Sea and the attitude in which the United States is placed by the Arbitration Commission selected to settle the seal question. In his estimation the decision of the Commission was flagrantly unjust, and that will be the opinion of all who read the author's learned arguments.

It is impossible, within the limits of this article, to detail all the valuable material in this book. Suffice it to say that it gives a very good idea of the nature of the country, its climate, its animals and plants. What has been discovered in the way of minerals up to the time of publishing the book, at the beginning of this year, is set forth, and so the hardy adventurer who goes to this far-off land will need the book as a help in his wanderings.

